# NT-proBNP Is a Predictor of Mortality in Adults with Pulmonary Arterial Hypertension Associated with Congenital Heart Disease

**DOI:** 10.3390/jcm12093101

**Published:** 2023-04-24

**Authors:** Susanne J. Maurer, Veronika Habdank, Jürgen Hörer, Peter Ewert, Oktay Tutarel

**Affiliations:** 1 Department of Congenital Heart Disease and Paediatric Cardiology, German Heart Centre Munich, TUM School of Medicine, Technical University of Munich, 80636 Munich, Germany; 2 Department of Congenital and Paediatric Heart Surgery, German Heart Centre Munich, Technical University of Munich, 80636 Munich, Germany; 3 Division of Congenital and Paediatric Heart Surgery, University Hospital Munich, Ludwig-Maximilians Universität, 81377 Munich, Germany; 4 DZHK (German Centre for Cardiovascular Research), Partner Site Munich Heart Alliance, 80992 Munich, Germany

**Keywords:** adult congenital heart disease, pulmonary hypertension, outcome

## Abstract

Background: About 5–10% of adults with congenital heart disease (ACHD) will develop pulmonary arterial hypertension (PAH), which is associated with significant mortality. Studies on risk factors for poor outcome in a contemporary cohort of these patients with PAH associated with CHD (PAH-CHD) are rare. Methods: In this retrospective, single-center study, adult patients with the diagnosis PAH-CHD who had at least one contact as an outpatient or inpatient at the German Heart Centre Munich during the period January 2010–September 2019 were included. Patients with PAH without a CHD were excluded. The primary endpoint was all-cause mortality. Results: Altogether, 158 patients (mean age 39.9 ± 15.4 years, female 64.6%) were included in the study. A pre-tricuspid shunt was present in 17.7%, other shunts in 51.3%, PAH associated with complex CHD in 22.8%, and segmental PAH in 8.2%. An NT-proBNP measurement at baseline was available in 95 patients (60.1%). During a median follow-up of 5.37 years [IQR 1.76–8.63], the primary endpoint occurred in 10 patients (6.7%). On univariate analysis, CRP (log) (HR 3.35, 95% CI (1.07–10.48), *p* = 0.037), NT-proBNP (log) (HR: 7.10, 95% CI: 1.57–32.23, *p* = 0.011), and uric acid (HR: 1.37, 95% CI: 1.05–1.79, *p* = 0.020) were predictors of the primary endpoint. On multivariate analysis, only NT-proBNP (log) (HR: 6.91, 95% CI: 1.36–35.02, *p* = 0.0196) remained as an independent predictor. Conclusion: NT-proBNP is an independent predictor of all-cause mortality in a contemporary cohort of PAH-CHD patients. The role of CRP and uric acid should be further assessed in future studies.

## 1. Introduction

Tremendous advances in the treatment of children with congenital heart disease (CHD) have been made in the fields of pediatric cardiology, cardiovascular surgery, and intensive care medicine in the last decades. As a consequence, nowadays it is expected that more than 90% of children with CHD will reach adulthood [1]. However, a cure is seldom achieved and, in the longer term, complications and sequela that require medical attention and therapy are common. Hence, a new patient group has emerged, adults with congenital heart disease (ACHD). One of the most severe sequelae in ACHD patients is the development of pulmonary arterial hypertension (PAH). It is estimated that about 5–10% of ACHD patients will develop PAH [2], which is associated with significant morbidity and mortality [3,4]. Early risk stratification is of importance and, therefore, there is an imminent need to identify risk factors for an unfavorable outcome in these PAH associated with CHD (PAH-CHD) patients. Several studies have already been conducted; however, risk factors for an unfavorable prognosis were mainly assessed in Eisenmenger syndrome patients. The parameters identified as predictors include brain natriuretic peptide (BNP) levels, the presence of a pre-tricuspid shunt or the performance on a six-minute walk distance test [5,6,7]. However, PAH-CHD is not limited to patients with Eisenmenger syndrome but does also include patients after defect closure as well as in combination with more complex CHD such as transposition of the great arteries or even segmental PAH as a consequence of major aorto-pulmonary collateral arteries and some of these types of PAH-CHD probably carry an even worse prognosis than Eisenmenger syndrome patients [8,9]. Studies analyzing risk factors for a worse clinical outcome that include the latter groups of PAH-CHD patients are scarce. However, we will likely see an increase in these patients with PAH-CHD who are not Eisenmenger syndrome patients, which is also likely to continue.

Therefore, the aim of our study was to describe risk factors for an unfavorable outcome in a contemporary cohort of PAH-CHD patients, including all variants.

## 2. Materials and Methods

This investigation was conducted as a retrospective, single-center study. All adult patients (>18 years of age) with the diagnosis PAH-CHD who had at least one contact as an outpatient or inpatient at the German Heart Centre Munich during the study period January 2010–September 2019 were included. Patients with PAH without an underlying diagnosis of CHD were excluded.

The start of follow-up was the first contact during the study period, while the end of the follow-up period was either the last contact during the study period or the date of death if the patient died during the study period.

As previously described, PAH-CHD was established and confirmed by echocardiography, cardiac MRI, and/or cardiac catheterization [10]. The underlying CHD was classified into several groups as follows: (1) pre-tricuspid shunts: atrial septal defects (ASD), anomalous pulmonary venous drainage; (2) other shunt lesions: ventricular septal defects (VSD), atrioventricular septal defects (AVSD), persistent ductus arteriosus, aortopulmonary window; (3) complex: PAH associated with complex CHD, for example, transposition of the great arteries after atrial switch surgery with a remaining VSD; and (4) segmental: PAH due to major aorto-pulmonary collateral arteries. Treatment followed the recommendations from guidelines [11,12].

Demographic data and information on medical as well as surgical history were retrieved from hospital records. The New York Heart Association classification (NYHA) was used to assess symptomatic status. Left and right ventricular systolic function was graded based on the results of routine transthoracic echocardiograms as normal, mildly, moderately or severely impaired, as previously described [13]. Arrhythmias encompassed any type of supraventricular or ventricular arrhythmia requiring treatment.

Laboratory values retrieved from medical records at the time of the baseline visit included hemoglobin, hematocrit, N-terminal pro-B type natriuretic peptide (NT-proBNP), C-reactive protein (CRP), creatinine, uric acid, and bilirubin.

The primary end-point was all-cause mortality. Cause of death was retrieved from hospital records.

Statistical analyses were performed with the software SPSS version 28 (IBM Corp., Armonk, NY, USA) and MedCalc version 20.210 (MedCalc Software, Mariakerke, Belgium). Continuous variables are presented as mean ± standard deviation or median (interquartile range) and categorical variables as number (percentage). The Mann–Whitney U test or Student’s *t*-test was used for comparison between groups for continuous variables, while the Chi-square test was used for categorical variables. The association between variables and the primary endpoint was assessed with univariate Cox proportional hazards analysis. Significant variables (*p* < 0.05) from the univariate analysis were subsequently included in a multivariate model in a stepwise fashion. Cut-off values for significant variables from the multivariate Cox proportional hazards analysis were derived using the Youden index with a receiver operating characteristics (ROC) curve. Kaplan–Meier curves and log-rank tests were used to compare survival (all-cause mortality) between patients with values below and above the cut-off of significant variables. All tests were performed two-sided and, for all analyses, a *p*-value < 0.05 was considered statistically significant.

## 3. Results

Altogether, 158 patients (mean age 39.9 ± 15.4 years, female 64.6%) were included in the study. A pre-tricuspid shunt was present in 17.7%, other shunts in 51.3%, PAH associated with complex CHD in 22.8%, and segmental PAH in 8.2%. Out of the 158 patients, 85 (53.8%) were on advanced PAH therapies at baseline. More information regarding the baseline characteristics is presented in Table 1. Nine patients did not have a follow-up appointment.

At least one hospitalization occurred in 80 patients (53.7%). Out of these, 29 were unplanned/emergency admissions. The reasons for these unplanned hospitalizations were worsening heart failure in 14, arrhythmias in 13, chest pain in one and infection in another one.

During a median follow-up of 5.37 years [IQR 1.76–8.63], the primary endpoint occurred in 10 patients (6.7%). The cause of death was cardiac in four patients, septicemia in three, and unknown in the remaining three. When comparing patients that have died during the study period with those alive at the end of follow-up regarding their baseline characteristics, patients who died were more likely to be in NYHA class IV (Table 1).

The univariate analysis for the whole cohort revealed no significant predictor for the primary endpoint (Table 2).

However, laboratory values were not included in this analysis because they were not available for the whole cohort. An NT-proBNP measurement at baseline was available in 95 patients (60.1%). The primary endpoint occurred in 7 (7.4%) out of these 95 patients. There was no significant difference between patients with an NT-proBNP measurement at baseline and those without regarding age (*p* = 0.090), sex (*p* = 0.429), type of congenital heart defect (*p* = 0.436), presence of Down syndrome (*p* = 0.987), follow-up duration (*p* = 0.182), or mortality (*p* = 0.510). Additionally, there was also no significant difference for NT-proBNP between patients with pre-tricuspid shunts (1868 ± 1875 ng/L), other shunt lesions (1219 ± 2430 ng/L), and PAH-CHD associated with complex CHD (1618 ± 1955 ng/L, *p* = 0.091). Therefore, a second analysis regarding the predictors of the primary end-point was carried out solely in the cohort with an available NT-proBNP measurement at baseline. A comparison of laboratory values between those patients in whom the primary endpoint occurred and those in which it did not is presented in Table 3. Deceased patients had a higher level of NT-proBNP (*p* = 0.022), CRP (*p* = 0.019), and uric acid (*p* = 0.010) at baseline.

On univariate analysis, CRP (log) (HR 3.35, 95% CI (1.07–10.48), *p* = 0.037), NT-proBNP (log) (HR: 7.10, 95% CI: 1.57–32.23, *p* = 0.011), and uric acid (HR: 1.37, 95% CI: 1.05–1.79, *p* = 0.020) were predictors of the primary endpoint (Table 4). On multivariate analysis, only NT-proBNP (log) (HR: 6.91, 95% CI: 1.36–35.02, *p* = 0.0196) remained as an independent predictor in the model (Table 4).

**Figure 1:**
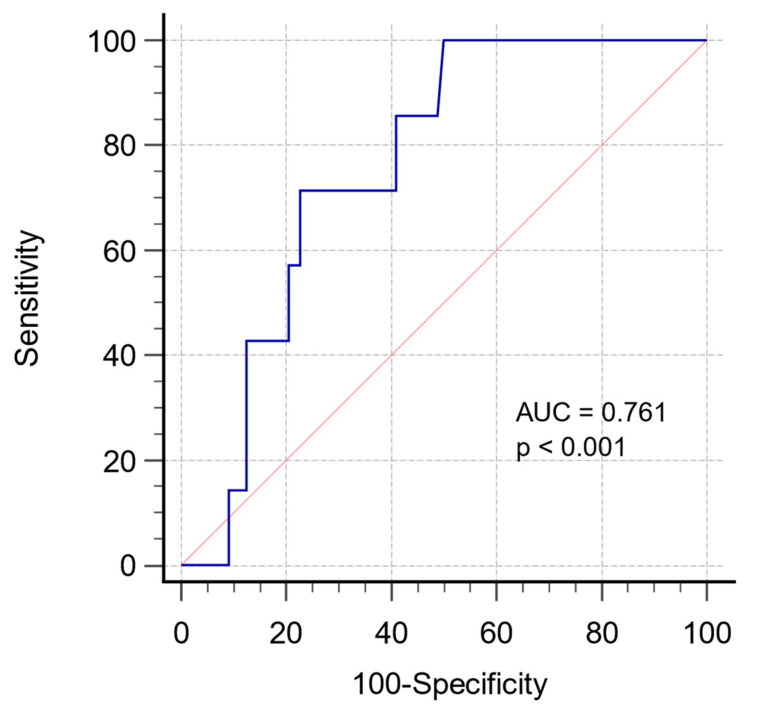
Receiver operating characteristics (ROC) curve for NT-proBNP as predictor of mortality.

The area under the receiver operating characteristics curve for NT-proBNP was 0.761 (Figure 1). The cut-off value for NT-proBNP was 538 ng/L.

**Figure 2:**
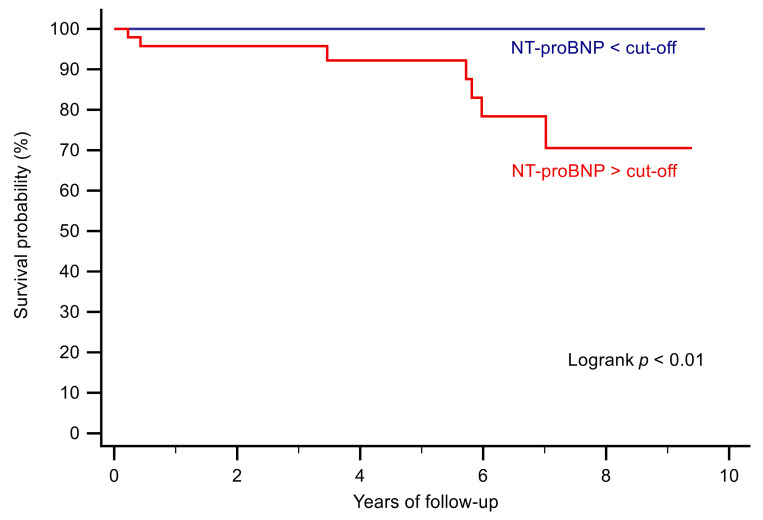
Kaplan–Meier curves stratifying patients according to an NT-proBNP value above or below the cut-off value.

In patients in whom NT-proBNP was above the cut-off value, survival was diminished (*p* < 0.01; Figure 2).

## 4. Discussion

During a median follow-up of 5.3 years, 6.7% of PAH-CHD patients died. NT-proBNP was the only independent predictor of mortality in this study. Furthermore, PAH-CHD patients were burdened with significant morbidity leading to hospitalization in more than 50% of patients and unplanned/emergency hospitalization in around 20% of patients.

Brain natriuretic peptides (BNP or NT-proBNP) are established biomarkers that are utilized in various forms of cardiovascular diseases, especially for the diagnosis of heart failure [14]. They can be used for prognostication and may also guide the treatment of heart failure [14]. Furthermore, their prognostic value in a large cohort of ACHD patients with a variety of underlying CHD has been investigated [15]. However, specific data for PAH-CHD patients were not reported in this study. Brain natriuretic peptides also play an important role in the assessment of PAH patients of various etiologies. Several risk stratification tools have been developed for PAH patients, for example, the score based on the “Registry to Evaluate Early and Long-term Pulmonary Arterial Hypertension Disease Management” (REVEAL) [16]. The plasma levels of BNP or NT-proBNP are an essential part of all of these risk stratification tools [17]. The prognostic value of brain natriuretic peptides in patients with PAH was also assessed in a recent systematic review and meta-analysis [17]. Overall, Hendriks and colleagues included 16 studies representing altogether 6999 patients with group 1 PAH in their analysis. They showed that increased levels of BNP or NT-proBNP were associated with a significantly increased risk of mortality or lung transplantation [17]. Accordingly, current European guidelines for the diagnosis and treatment of pulmonary hypertension recommend using BNP or NT-proBNP during screening for PAH and the diagnostic work-up to establish the diagnosis [18]. Furthermore, they are part of a comprehensive risk assessment tool, the three-strata model, which is recommended in these guidelines [18]. However, most of the studies that established the different risk stratification tools include only a small number of PAH-CHD patients. For example, the validation cohort of the REVEAL score had only 4.8% of PAH-CHD patients, while their number was 11.8% in the development cohort [16]. Therefore, their applicability to PAH-CHD patients needs to be assessed. In a large single-center study in patients with Eisenmenger syndrome from the Royal Brompton Hospital in London/UK, BNP concentrations predicted survival independent of renal function, the presence of Down syndrome, or the achieved six-minute walk distance [5]. The results of the current study, which confirm NT-proBNP as an independent predictor of all-cause mortality, are in accordance with these findings and expand them to the whole cohort of PAH-CHD patients including other forms of PAH-CHD in addition to Eisenmenger syndrome patients. Furthermore, they are also in accordance with a previous report from our group, in which NT-proBNP was an independent predictor of all-cause mortality in PAH-CHD patients over the age of 40 years [10]. Hence, BNP or NT-proBNP plays an important role in the assessment and risk stratification of PAH-CHD patients. However, they should only be a part of a multidimensional risk assessment as previously proposed [18,19], which should, for example, also include parameters such as functional class and the results of a six-minute walk test.

Interestingly, CRP was a predictor of all-cause mortality in the univariate analysis. CRP is an acute-phase protein that is synthesized by hepatocytes in response to cytokines and functions as an easily available and widely used marker to assess inflammation in clinical practice [2]. Its association with increased cardiovascular risk in healthy subjects and also patients with various established cardiovascular diseases has been shown [2]. In two large cohorts of ACHD patients with a variety of underlying CHD, it provided additional prognostic value [20,21]. However, data that are specific to PAH-CHD patients were not reported separately in either study. Importantly, in patients with idiopathic PAH or chronic thromboembolic pulmonary hypertension, CRP correlated with NYHA class, right atrial pressure, and the six-minute walking test distance [22]. Furthermore, it was also significantly higher in non-survivors compared to survivors [22]. In patients with PAH-CHD, a previous study from the UK assessed CRP as a predictor of outcome [2]. The authors were able to show that CRP was a strong predictor of mortality independent of age, the presence of Down syndrome, or the use of advanced PAH therapies [2]. In this study, Scognamiglio and colleagues reported an optimal cut-off value for CRP of 10 mg/L and showed that patients with CRP values above this threshold had a threefold increased risk of death [2]. Notably, this study included all various types of PAH-CHD patients and did not rely solely on patients with Eisenmenger syndrome [2]. Their results are also in accordance with the findings of the current study, which identified CRP as a predictor of all-cause mortality in the univariate analysis. However, in the multivariate analysis, only NT-proBNP remained as an independent predictor. Unfortunately, the measurements of BNP or NT-proBNP were not reported in the study by Scognamiglio and colleagues [2]. Therefore, the jury might still be out regarding the role of CRP as an independent predictor of mortality in PAH-CHD patients. However, the concept of taking a look from a different pathophysiological angle at the disease process of PAH-CHD patients in addition to NT-proBNP, which mostly reflects the hemodynamic burden [2], seems to be reasonable and promising.

Uric acid was also a predictor of outcome in the univariate analysis. Uric acid, the final product of purine metabolism, is gaining increasing importance in the evaluation of cardiovascular disease [23]. It is highly related to inflammation and oxidative stress due to a proposed mechanism of inhibition of endothelial nitric oxide and vascular inflammation [23]. Furthermore, in patients with various types of PAH, uric acid has been described as a marker of disease progression as well as treatment response and survival and correlates with hemodynamic abnormalities [24,25,26]. In PAH-CHD patients, a recent study by Chiu and colleagues in 190 patients with PAH-CHD out of a total cohort of 4301 ACHD patients reported uric acid as a predictor of poor long-term outcome [27]. Interestingly, NT-proBNP was a predictor of mortality in the univariate model, but not in the multivariable model in this study [27]. A possible explanation for this finding is provided by the study authors themselves, emphasizing that not all patients had data regarding NT-proBNP, which might have affected its statistical power [27]. Further studies are needed and warranted to elucidate the role of uric acid in PAH-CHD patients, which might also provide us with a different pathophysiological angle on the disease process of these patients.

Other predictors of outcome besides laboratory parameters have been identified in previous studies of PAH-CHD patients. Moceri et al. assessed echocardiographic predictors of outcome in patients with Eisenmenger syndrome, excluding those with complex CHD [28]. They included 181 patients and generated a composite score based on the strongest echocardiographic predictors of mortality [28]. It included a tricuspid annular plane systolic excursion (TAPSE) below 15 mm, a ratio of right ventricular effective systolic to diastolic duration ≥ 1.5, a right atrial area ≥ 25 cm^2^, and a ratio of right atrial to left atrial area ≥ 1.5 [28]. This composite score was strongly related to mortality and the area under the curve on the receiver operating curve analysis was 0.90. Interestingly, the predictive power of the composite score did not improve with the inclusion of BNP [28]. Unfortunately, the measurements of these echocardiographic parameters were not available for the patients of the current study cohort. They should be included in the assessment of PAH-CHD patients in future studies. However, we have to keep in mind that it is unclear whether these echocardiographic predictors and the composite score are also valid for patients with complex CHD because of their exclusion from the study of Moceri and colleagues [28].

Kempny and colleagues assessed whether the six-minute walk test distance was predictive of the outcome of mortality in Eisenmenger syndrome patients [6]. Altogether, 210 patients under active follow-up between 2000 and 2012 at the Royal Brompton Hospital were included. On time-dependent Cox analysis, the six-minute walk test distance and baseline oxygen saturation were predictors for death, while age and functional class were not [6]. Unfortunately, measurements of BNP or NT-proBNP were not reported by Kempny and colleagues. In the cohort of the current study, an assessment of the six-minute walk test distance at baseline was only available in approximately 21% of the patients. Therefore, it was not included in the statistical analysis. Recapitulating the results of all previous studies together with the results of the current investigation, a study with a comprehensive assessment of all these different parameters combined, including different laboratory values, imaging parameters derived from echocardiography as well as parameters from exercise tests, such as the six-minute walk test distance, seems to be warranted. Furthermore, all types of PAH-CHD should be included in such a study. The aim would be to investigate all of the various variables, which were predictive of a worse outcome in previous studies, in a direct comparison with each other. This could lead to a final composite score, which could then be used in daily clinical practice for the assessment and risk stratification of PAH-CHD patients.

The mortality of the PAH-CHD cohort in the study from Scognamiglio et al. was higher (22%) compared to the mortality in the current cohort of 6.7% [2]. The mortality in the current cohort was also lower than the reported number of 25% from a multicenter study of Eisenmenger patients [7]. While we can only speculate about the reasons for this finding, it is noteworthy that the UK study included patients from 2000 to 2012, while the multicenter study covered the period of 2000–2015 [2,7]. In contrast, our study period was 2010–2019, reflecting a more contemporary cohort. Additionally, the multicenter study included only patients with Eisenmenger syndrome, who might have a different disease trajectory than the whole PAH-CHD cohort [27]. Furthermore, patients in our study were more often on advanced PAH therapies at baseline (53.8%) than in the study by Scognamiglio et al. (13%) [2]. While our study was not designed to assess the impact of these advanced PAH therapies on the outcome of patients, there is a strong signal from a previous study by Dimopoulos et al. that these are indeed beneficial for PAH-CHD patients [29]. However, a double-blind randomized controlled trial of advanced PAH therapies in patients with PAH-CHD using mortality as the primary endpoint is still lacking.

A limitation of our study is its retrospective nature with the associated obvious drawbacks inherent to this type of study. For example, variables such as NT-proBNP were not available in all patients. Additionally, the objective measures of exercise capacity such as the six-minute walk test were not regularly performed. Furthermore, the cause of death was not available in three patients.

## 5. Conclusions

In conclusion, NT-proBNP is an independent predictor of all-cause mortality in a contemporary cohort of PAH-CHD patients. The role of CRP and uric acid in combination with other prognostic parameters should be further assessed in future studies.

## Figures and Tables

**Table 1:** Baseline characteristics.

	All	Alive	Dead	*p*
N	158	148	10	
Age in years	39.9 ± 15.4	39.9 ± 15.7	40.2 ± 10.7	0.76
Female, n (%)	102 (64.6)	95 (64.2)	7 (70.0)	0.71
Down syndrome	30 (19.0)	27 (18.2)	3 (30.0)	0.36
Congenital heart defect, n (%)				0.56
Pre-tricuspid shunt	28 (17.7)	27 (18.2)	1 (10.0)	
Other shunts	81 (51.3)	77 (52.0)	4 (40.0)	
Complex	36 (22.8)	32 (21.6)	4 (40.0)	
Segmental	13 (8.2)	12 (8.1)	1 (10.0)	
Previous cardiac surgery	71 (44.9)	66 (44.6)	5 (50.0)	0.77
History of, n (%)				
Atrial arrhythmias	40 (25.5)	38 (25.9)	2 (20.0)	0.68
Hemoptysis	11 (7.0)	10 (6.8)	1 (10.0)	0.70
Thromboembolic events	22 (14.1)	19 (13.0)	3 (30.0)	0.14
Endocarditis	4 (2.5)	4 (2.7)	0	0.60
Brain abscess	3 (1.9)	3 (2.1)	0	0.65
Pericardial effusion	5 (3.5)	4 (3.0)	1 (10.0)	0.25
Advanced PAH therapies	85 (53.8)	79 (53.4)	6 (60.0)	0.68
Monotherapy	70 (44.3)	64 (43.2)	6 (60.0)	0.64
Dual therapy	14 (8.9)	14 (9.5)	0	
Triple therapy	1 (0.6)	1 (0.7)	0	
NYHA class, n (%)				<0.05
I	18 (11.4)	17 (11.5)	1 (10.0)	
II	62 (39.2)	58 (39.2)	4 (40.0)	
III	62 (39.2)	60 (40.5)	2 (20.0)	
IV	12 (7.6)	9 (6.1)	3 (30.0)	
Missing	4 (2.5)	4 (2.7)	0	
LV function, n (%)				0.15
Normal	116 (73.4)	109 (73.6)	7 (70.0)	
Mild	7 (4.4)	5 (3.4)	2 (20.0)	
Moderate	8 (5.1)	7 (4.7)	1 (10.0)	
Severe	3 (1.9)	3 (2.0)	0	
Missing	24 (15.2)	24 (16.2)	0	
RV function, n (%)				0.10
Normal	104 (65.8)	100 (67.6)	4 (40.0)	
Mild	8 (5.1)	7 (4.7)	1 (10.0)	
Moderate	16 (10.1)	13 (8.8)	3 (30.0)	
Severe	4 (2.5)	4 (2.7)	0	
Missing	26 (16.5)	24 (16.2)	2 (20.0)	
Cardiovascular risk factors				
Smoking, n (%)	5 (3.2)	5 (3.4)	0	0.75
Diabetes, n (%)	9 (5.7)	9 (6.1)	0	0.42
Arterial hypertension, n (%)	29 (18.4)	29 (19.6)	0	0.12
Hypercholesterolemia, n (%)	6 (3.8)	6 (3.8)	0	0.52

LV: left ventricular; NYHA: New York Heart Association; PAH: pulmonary arterial hypertension; RV: right ventricular.

**Table 2:** Univariate analysis of predictors for all-cause mortality for the whole cohort.

	Univariate	
Variable	HR (95% CI)	*p*
Age	1.02 (0.98–1.07)	0.38
Male	0.65 (0.17–2.52)	0.53
Reduced LV function	3.30 (0.85–12.81)	0.08
NYHA functional class	1.99 (0.89–4.41)	0.09
Reduced RV function	3.06 (0.86–10.86)	0.08
Down syndrome	1.64 (0.42–6.35)	0.47

HR: hazard ratio; NYHA: New York Heart Association; LV: left ventricular; RV: right ventricular.

**Table 3:** Laboratory values for cohort with NT-proBNP measurement at baseline.

	Alive	Dead	*p*
Hemoglobin, g/dL	17.3 ± 3.9	19.9 ± 4.1	0.10
Hematocrit, %	51.5 ± 10.9	57.1 ± 11.1	0.19
NT-proBNP, ng/L	1450 ± 2267	2422 ± 1399	0.022
C-reactive protein, mg/L	6.7 ± 13.2	14.8 ± 12.2	0.019
Creatinine, mg/dL	0.93 ± 0.28	0.91 ± 0.09	0.72
Uric acid, mg/dL	6.9 ± 2.3	9.5 ± 2.1	0.010
Bilirubin, mg/dL	0.99 ± 0.69	1.22 ± 0.62	0.23

**Table 4:** Univariate and multivariate analysis of predictors for all-cause mortality with NT-proBNP.

	Univariate		Multivariate	
Variable	HR (95% CI)	*p*	HR (95% CI)	*p*
Age	1.02 (0.97–1.08)	0.46		
Male	1.04 (0.23–4.67)	0.96		
Reduced LV function	4.03 (0.90–18.11)	0.07		
NYHA functional class	1.82 (0.67–4.91)	0.24		
Reduced RV function	4.06 (0.91–18.18)	0.07		
Down syndrome	1.24 (0.24–6.42)	0.80		
Creatinine (log)	8.03 (0.01–7423)	0.55		
Pre-tricuspid shunt	0.83 (0.10–7.23)	0.87		
**CRP (log)**	**3.35 (1.07–10.48)**	**0.037**		
**NT-proBNP (log)**	**7.10 (1.57–32.23)**	**0.011**	**6.91 (1.36–35.02)**	**0.0196**
**Uric acid**	**1.37 (1.05–1.79)**	**0.020**		

CRP: C-reactive protein; HR: hazard ratio; NYHA: New York Heart Association; LV: left ventricular; RV: right ventricular. Significant results are in bold font.

## Data Availability

The data underlying this article cannot be shared publicly due to data privacy reasons and the according German regulations.
